# Diatoms from Wrangell-St. Elias National Park, Alaska, USA

**DOI:** 10.3897/phytokeys.113.29456

**Published:** 2018-12-06

**Authors:** Loren Bahls, Tara Luna

**Affiliations:** 1 Montana Diatom Collection, Helena, MT 59601 USA Montana Diatom Collection Helena United States of America; 2 East Glacier Park, Montana, 59434 USA Montana Diatom Collection Helena United States of America

**Keywords:** diatoms, Alaska, diatom biodiversity, diatom biogeography, glacier diatoms

## Abstract

As a contribution to our knowledge of diatom biodiversity and biogeography in the United States, high resolution light microscope images are provided for 139 diatom taxa recorded from lake, stream, spring and glacier habitats in Wrangell-St. Elias National Park, Alaska. The spring had the highest taxa richness of the four habitats that were sampled, likely owing to the relative stability of this habitat compared to the others. Most of the taxa were described from northern and alpine locations in Europe and North America and are typical of habitats in the northern Rocky Mountains, with two notable exceptions. *Surirellaarctica* had been reported previously only from locations in the High Arctic of North America, north of 68°N latitude. *Gomphonemacaperatum* has a disjunct distribution in montane regions of the eastern and far western contiguous United States. This may be the first record of this taxon from Alaska.

## Introduction

For a land area as large as Alaska (1,717,856 km^2^), there are relatively few published articles on freshwater diatom taxonomy and biodiversity (Patrick and Freese 1961, Foged 1971, 1981, McLaughlin and Stone 1986, Hein 1990). All of these studies predate the general availability of scanning electron microscopy and relied on taxonomic references that today are widely considered to be incomplete and out-of-date. One recent study (Pite et al. 2009) addressed the historical morphology and abundance of two *Didymosphenia* species in an Alaskan Lake.

**Figure 1.**
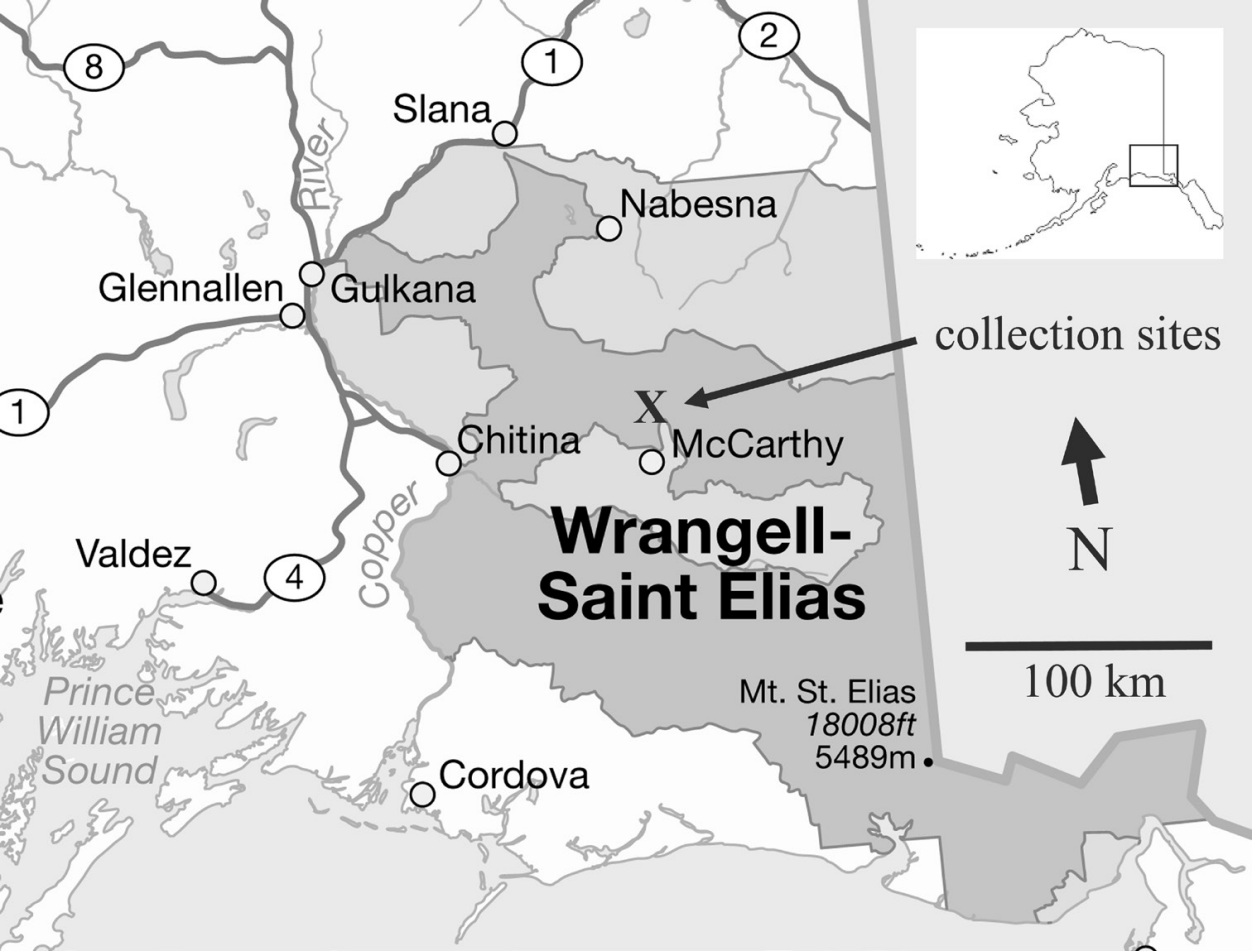
Map of Wrangell-St. Elias National Park showing approximate location of collection sites. Base map: U. S. National Park Service.

Wrangell-St. Elias National Park and Preserve (WRST) is located in the southeast corner of Alaska (Fig. 1). At 53,320 km^2^, it is the largest national park in the United States, six times larger than Yellowstone and about the same size as the country of Croatia. It is also one of the least visited of the national parks and much of it is untracked wilderness. Elevations in the park range from sea level to 5,489 m Mt. St. Elias. The mountainous terrain and ample winter snowfall from north Pacific weather systems produce some of the largest glaciers and ice fields in the world (US National Park Service 2018).

Although diatoms have been used to assess water quality and climate change within and near WRST (Simmons 2007, Brabets et al. 2011, Griffiths 2015), these studies did not include images of voucher specimens to verify identifications. Published studies on diatom taxonomy and biodiversity in the park appear to be wanting. Here we present high-resolution LM images of 139 diatom taxa collected in early summer 2018 from a lake, a stream, a spring and a glacier in WRST. This paper is intended only as a preliminary checklist of park diatoms with images of voucher specimens (illustrated checklist). Results are discussed briefly with respect to diatom biodiversity and biogeography.

## Methods

**Figures 2–4.**
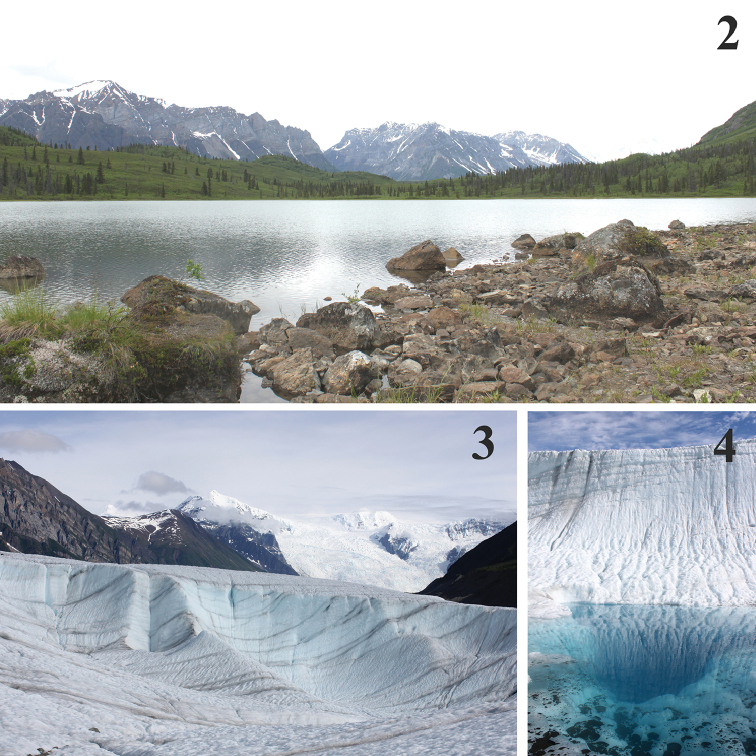
Collection sites. **2** Donoho Lake **3** Root Glacier **4** Pool of standing water (moulin) on Root Glacier. Photos: Tara Luna.

Samples of benthic diatoms were collected from four sites in WRST (Table 1, Fig. 1). Donoho Lake (Fig. 2) is a shallow lake surrounded by white and black spruce forest, located between the Kennicott and Root Glaciers south of Donoho Peak. Jumbo Creek is a high-gradient perennial stream that originates in the mountains east of Root Glacier and north of Kennecott Mine and Townsite. McCarthy Spring is located east of Kennicott River and serves as the water source for the town of McCarthy. Four samples were collected from Root Glacier (Figs 3, 4), one each from three open pools (moulins) on the Root Glacier Ice Field and one from melt water discharging from a smaller rock glacier on the ice field.

**Table 1.** Samples collected on 29 June 2018 from WRST, Alaska. Sample numbers are for the Montana Diatom Collection and database. Slide numbers are for slides in the diatom collection at the University of Montana Herbarium, Missoula (MONTU). The numbers in the column headed “WRST” are National Park Service catalogue numbers for accession WRST-00483.

Sample number	Site name	Latitude (°N)	Longitude (°W)	Slide number	WRST
6955	Donoho Lake	61.5275, -142.9580		50–31	22949
6956	Jumbo Creek	61.5030, -142.8972		50–32	22950
6957	McCarthy Spring	61.4344, -142.9278		50–33	22951
6958	Root Glacier (pools)	61.5098, -142.9265		50–34	22952
6959	Root Glacier (rock glacier)	61.5122, -142.9211		50–35	22953

Substrata that were sampled at WRST collection sites were cobbles (Jumbo Creek), sediment (all sites) and peat (moulins). As there were very few diatom cells in the samples from Root Glacier pools, they were combined for the purpose of sample processing and reporting. The pools appeared to extend to the bottom of the glacier, so diatoms in the pool samples may have originated from greater depth. The Root Glacier Ice Field is up to 213 m thick at this location, which is near its confluence with the Kennicott Glacier. Collection sites on the glacier were 6 km below the massive 2,133 m Staircase Ice Fall, at the head the valley, which is the largest icefall outside the Himalayas.

Sediment samples were collected with a large-bore pipette (5 mm diameter) with a suction bulb. The pipette was rinsed with ambient water twice between collection sites. Approximately 7.5 cm^3^ of water and sediment was pulled from the upper 1 cm of sediment at each sample site and stored in collection bottles. At Jumbo Creek, the surface film on several cobbles was scraped into the sample bottle and some peat material was included in the moulin samples. Iodine was added to each sample within 12 hours of collection. In the laboratory, samples were treated with 30% hydrogen peroxide (H_2_O_2_) and heated gently for several days to remove organic matter. After several rinses in distilled water, cleaned diatom material was dried on cover slips and mounted permanently on glass slides using Naphrax.

One slide per sample was examined under light microscopy (LM) with differential interference contrast optics and images were captured using a Leica DM LB2 research microscope and a Spot Insight monochrome digital camera (Model 14.0). Slides examined for this study will be deposited in the University of Montana Herbarium, Missoula (MONTU) on completion of the study. Imaged diatoms were identified to the lowest practical taxonomic unit using available identification resources, mainly Patrick and Reimer 1966, 1975, Krammer and Lange-Bertalot 1986, 1988, 1991a, 1991b, Krammer 1997a, 1997b, 2000, 2002, 2003, Lange-Bertalot 2001, Levkov 2009, Lange-Bertalot et al. 2011 and Levkov et al. 2013, 2016. The Diatoms of North America website (INSTAAR 2018) and other sources were also consulted as needed. The INA card file at the University of California Herbarium (2018) was consulted for type localities of taxa.

Each slide was systematically scanned at 100 magnifications to locate very large taxa that might otherwise be missed while scanning at higher magnifications. After these large taxa were identified, listed and photographed, slides were examined under oil immersion at 630 and 1,000 magnifications in order to find, identify and photograph smaller taxa. A “random walk” was taken around each slide and additional taxa were listed and photographed until no additional taxa were found after 20 minutes of scanning. The number of images that were captured of each taxon is roughly proportional to the relative abundance of that taxon in a sample.

## Results

A total of 139 taxa were identified at the genus or subgenus level (Table 2, Plates 1–14). None of the species is described as new to science, but some are designated as unknowns (e.g. *Hantzschia* sp.) or as comparable to another taxon (cf.). Alternate identifications are provided for some taxa in the plate legends.

**Table 2.** List of taxa and key to plates.

Taxa	Plate	Donoho Lake	Jumbo Creek	McCarthy Spring	Root Glacier	Type Locality
*Achnanthidiumgracillimum* (Meister) Lange-Bertalot	4		×			Japan
*Achnanthidiumminutissimum* (Kützing) Czarnecki	4	×	×	×		Germany
*Amphora* Ehrenberg in Kützing	10	×				Europe
*Amphoracopulata* (Kützing) Schoeman & Archibald	10		×			Germany
*Amphorainariensis* Krammer	10		×	×		Finnish Lapland
*Amphorapediculus* (Kützing) Grunow	10			×		Germany
*Brachysiramicrocephala* (Grunow) Compére	6		×			Austria
*Caloneisalpestris* (Grunow) Cleve	7	×		×		Austria
*Caloneisfalcifera* Lange-Bertalot, Genkal & Vekhov	7	×				Russia
*Caloneissilicula* (Ehrenberg) Cleve	7			×		New England, USA
*Caloneistenuis* (Gregory) Krammer	7			×		Scotland
*Caloneisthermalis* (Grunow) Krammer	7	×				Germany
*Cocconeisplacentula* Ehrenberg	4		×	×	×	Germany
*Cymatopleurasolea* (Brébisson) W. Smith	13			×		France
*Cymbellaalpestris* Krammer	9	×		×		Switzerland
*Cymbellacleve-eulerae* Krammer	9		×			Sweden
*Cymbellacosleyi* Bahls	9		×			Montana, USA
*Cymbellaexcisiformis* Krammer	9				×	Germany
Cymbellaneocistulavar.neocistula Krammer	9		×	×		Germany
Cymbellaneocistulavar.islandica Krammer	9		×			Iceland
*Cymbopleuraangustata* (W. Smith) Krammer	9	×			×	Scotland
*Cymbopleuraaustriaca* (Grunow) Krammer	9			×		Austria
*Cymbopleuraincerta* (Grunow) Krammer	9		×	×		Norway
*Cymbopleuralapponica* (Grunow) Krammer	9		×		×	Swedish Lapland
*Cymbopleuranaviculiformis* (Auerswald) Krammer	9				×	Germany
*Cymbopleuraoblongata* Krammer	9		×		×	Spitsbergen
*Cymbopleurasubaequalis* (Grunow) Krammer	9				×	Belgium
*Delicata* Krammer	10		×			France
*Delicataalpestris* (Krammer) Bahls	10		×			Austria
*Delicatadelicatula* (Kützing) Krammer	10		×			France
*Denticulakuetzingii* Grunow	11	×		×	×	Austria
*Denticulatenuis* Kützing	11			×		Germany
*Diatomatenuis* Agardh	1				×	Scandinavia
*Diatomavulgaris* Bory de Saint-Vincent	1	×				France
*Diploneiskrammeri* Lange-Bertalot & Reichardt	6			×		Austria
*Encyonemaneogracile* Krammer	10				×	Finnish Lapland
*Encyonemaperminutum* Krammer	10		×			Spitsbergen
*Encyonemasilesiacum* (Bleisch) Mann	10			×	×	Germany
*Encyonopsisalpina* Krammer & Lange-Bertalot	10		×			Germany
*Encyonopsiscesatii* (Rabenhorst) Krammer	10			×		Italy
*Encyonopsisczarneckii* Bahls	10		×			Montana, USA
*Encyonopsismontana* Bahls	10		×			Montana, USA
*Encyonopsisstafsholtii* Bahls	10		×			Montana, USA
*Encyonopsissubminuta* Krammer & Reichardt	10		×			Switzerland
*Eucocconeisalpestris* (Brun) Lange-Bertalot	4		×	×		Switzerland
*Eucocconeisflexella* (Kützing) Meister	4		×	×		Switzerland
*Eucocconeislaevis* (Østrup) Lange-Bertalot	4		×	×		Denmark
*Eunotiaarcus* Ehrenberg	4			×		Sweden
*Eunotiavalida* Hustedt	4		×			Switzerland
*Fragilaria* Lyngbye	2		×	×		Russia?
*Fragilariaamphicephala* Ehrenberg	2			×		Oregon, USA
*Fragilariasepes* Ehrenberg	2	×				Russia
*Fragilariatenera* (W. Smith) Lange-Bertalot	2		×			Ireland
*Fragilariavaucheriae* (Kützing) Petersen	2			×		Germany
*Frustuliaamosseana* Lange-Bertalot in Rumrich et al.	2	×				Scotland
*Gomphonema* Agardh	8			×	×	Germany
*Gomphonemabozenae* Lange-Bertalot & Reichardt	8			×		Finland
*Gomphonemacaperatum* Ponader & Potapova	8				×	Virginia, USA
*Gomphonemalateripunctatum* Reichardt & Lange-Bertalot	8		×			Germany
*Gomphonemaminusculum* Krasske	8				×	Tristan da Cunha
*Gomphonemaolivaceoides* Hustedt	8				×	Germany
*Gomphonemapseudobohemicum* Lange-Bertalot & Reichardt	8				×	Germany
*Gomphonemapumilum* (Grunow) Reichardt & Lange-Bertalot	8		×			Belgium
*Gyrosigma* Hassall	6	×				Germany
*Hannaeaarcus* (Ehrenberg) Patrick	2		×			Germany
*Hantzschia* Grunow	12	×		×		USA
*Hantzschiaabundans* Lange-Bertalot	12				×	Germany
*Hantzschiaamphioxys* (Ehrenberg) Grunow	12			×	×	USA
*Hantzschiaelongata* (Hantzsch) Grunow	12	×				Germany
*Hantzschiahyperborea* (Grunow) Lange-Bertalot	12	×				Russia
*Hygropetrabalfouriana* (Grunow) Krammer	7			×		Scotland
*Kurtkrammeriaaequalis* (W. Smith) Bahls	10				×	Scotland
*Lindaviaantiqua* (W. Smith) Nakov et al.	1			×		Ireland
*Luticolamutica* (Kützing) Mann	6			×		Germany
*Luticolaventricosa* (Kützing) Mann	6			×		Germany
*Meridioncirculare* (Greville) Agardh	1		×			Scotland
*Muelleriagibbula* (Cleve) Spaulding & Stoermer	5	×				Norway
*Naviculaangusta* Grunow	7			×	×	Austria
*Naviculacryptocephala* Kützing	7	×				Germany
*Naviculacryptotenella* Lange-Bertalot	7	×				Belgium
*Naviculalanceolata* (Agardh) Ehrenberg	7				×	Germany
*Naviculalibonensis* Schoeman	7	×				Lesotho
*Navicularadiosa* Kützing	7			×		Germany
*Naviculaseibigiana* Lange-Bertalot	7		×			Switzerland
*Naviculasubconcentrica* Lange-Bertalot	7	×	×			Germany
*Naviculavulpina* Kützing	7		×	×		Germany
*Neidiomorphabinodiformis* (Krammer) Cantonati et al.	5			×		Germany
*Neidium* Pfitzer	5	×				Germany
*Neidiumbergii* (Cleve-Euler) Krammer	5	×				Scandinavia
*Neidiumbisulcatum* (Lagerstedt) Cleve	5	×				Spitsbergen
*Neidiumbobmarshallensis* Bahls	5	×				Montana, USA
*Neidiumdistinctepunctatum* Hustedt	5	×				Austria
*Neidiumfogedii* Bahls	5	×		×		Alaska, USA
Neidiumkozlowiivar.ellipticum Mereschkowsky	5	×			×	Tibet
*Nitzschiaalpina* Hustedt	11			×		Switzerland
*Nitzschiaamphibia* Grunow	11	×				Austria
*Nitzschiaangustata* (W. Smith) Grunow	11			×		Sussex, UK
*Nitzschiadissipata* (Kützing) Rabenhorst	11			×		Germany
*Nitzschiadissipata var. oligotraphenta* Lange-Bertalot	11				×	Austria
*Nitzschiaexilis* Sovereign	11	×				Oregon, USA
*Nitzschiafonticola* (Grunow) Grunow	11			×		Belgium
*Nitzschiahomburgiensis* Lange-Bertalot	11			×		Germany
*Nitzschiainconspicua* Grunow	11			×		Austria
*Nitzschialacuum* Lange-Bertalot	11		×			Germany
*Nitzschialinearis* W. Smith	11			×		UK
*Nitzschiapalea* (Kützing) W. Smith	11	×				Germany
*Nitzschiaperminuta* Grunow	11			×		unknown
*Nitzschiapura* Hustedt	11		×			Germany
*Nitzschiapusilla* (Kützing) Grunow	11		×			Germany
*Nitzschiasublinearis* Hustedt	11		×	×		Austria
*Odontidiumhyemale* Kützing	1		×	×	×	Germany
*Odontidiummesodon* (Ehrenberg) Kützing	1			×		Germany
*Pinnularia* Ehrenberg	8			×		Germany
*Pinnulariakrammeri* Metzeltin	8	×				Finland
*Pinnulariapermicrostauron* Krammer & Metzeltin	8	×				Finland
*Pinnulariasinistra* Krammer	8		×			Germany
*Pinnulariasubanglica* Krammer	8				×	Sweden
*Pinnulariasubcommutata* Krammer	8			×		Belgium
*Pinnulariaviridis* (Nitzsch) Ehrenberg	8	×				Germany
*Placoneisabiskoensis* Hustedt	6			×		Sweden
*Planothidiumlanceolatum* (Brébisson) Lange-Bertalot	4				×	France
*Psammothidiumhelveticum* (Hustedt) Bukhtiyarova & Round	4				×	Switzerland
*Pseudostaurosirarobusta* (Fusey) Williams & Round	3			×		France
*Rhopalodiagibba* (Ehrenberg) O. Müller	14	×				Siberia
*Sellaphoralaevissima* (Kützing) Mann	6			×		Italy
*Sellaphorapupula* (Kützing) Mereschkowsky	6			×		Germany
*Stauroneisamphicephala* Kützing	6	×				Germany
*Stauroneisgracilis* Ehrenberg	6	×				French Guiana
*Stauroneisreichardtii* Lange-Bertalot et al.	6	×				Italy
*Stauroneisvandevijveri* Bahls	6	×				Montana, USA
*Staurosira* Ehrenberg	3			×		Connecticut, USA
*Staurosiraconstruens* Ehrenberg	3			×		Connecticut, USA
Staurosiraconstruensvar.venter (Ehrenberg) Hamilton	3			×		Germany
*Staurosirellalapponica* (Grunow) Williams & Round	3			×		Sweden
*Staurosirellaleptostauron* (Ehrenberg) Williams & Round	3			×		Germany
*Staurosirellapinnata* (Ehrenberg) Williams & Round	3			×		USA
*Surirellaarctica* (Patrick & Freese) Veselá & Potapova	14	×				Alaska, USA
*Surirellaminuta* Brèbisson	14				×	France
*Ulnariaulna* (Nitzsch) Compère	2				×	Germany
**Total taxa**		39	41	61	29	

Most taxa were described from type material collected in Europe (117 taxa), mostly northern Europe (Table 2). Other type localities include North America (18 taxa) [including 2 taxa from Alaska], Africa (1), Asia (1), Japan (1), Tristan da Cunha (1) and unknown (1). The total number of taxa in each habitat ranged from 29 on Root Glacier to 61 in McCarthy Spring.

Except for McCarthy Spring, diatom cells were scarce in all of the samples, as may be expected for such austere habitats. Glacial sediment (rock flour) dominated all but the McCarthy Spring sample and often obscured specimens for photography. Capturing quality images of voucher specimens was further hindered by diatom frustules that were often broken, eroded or encrusted with lime.

## Plates

**Plate 1.**
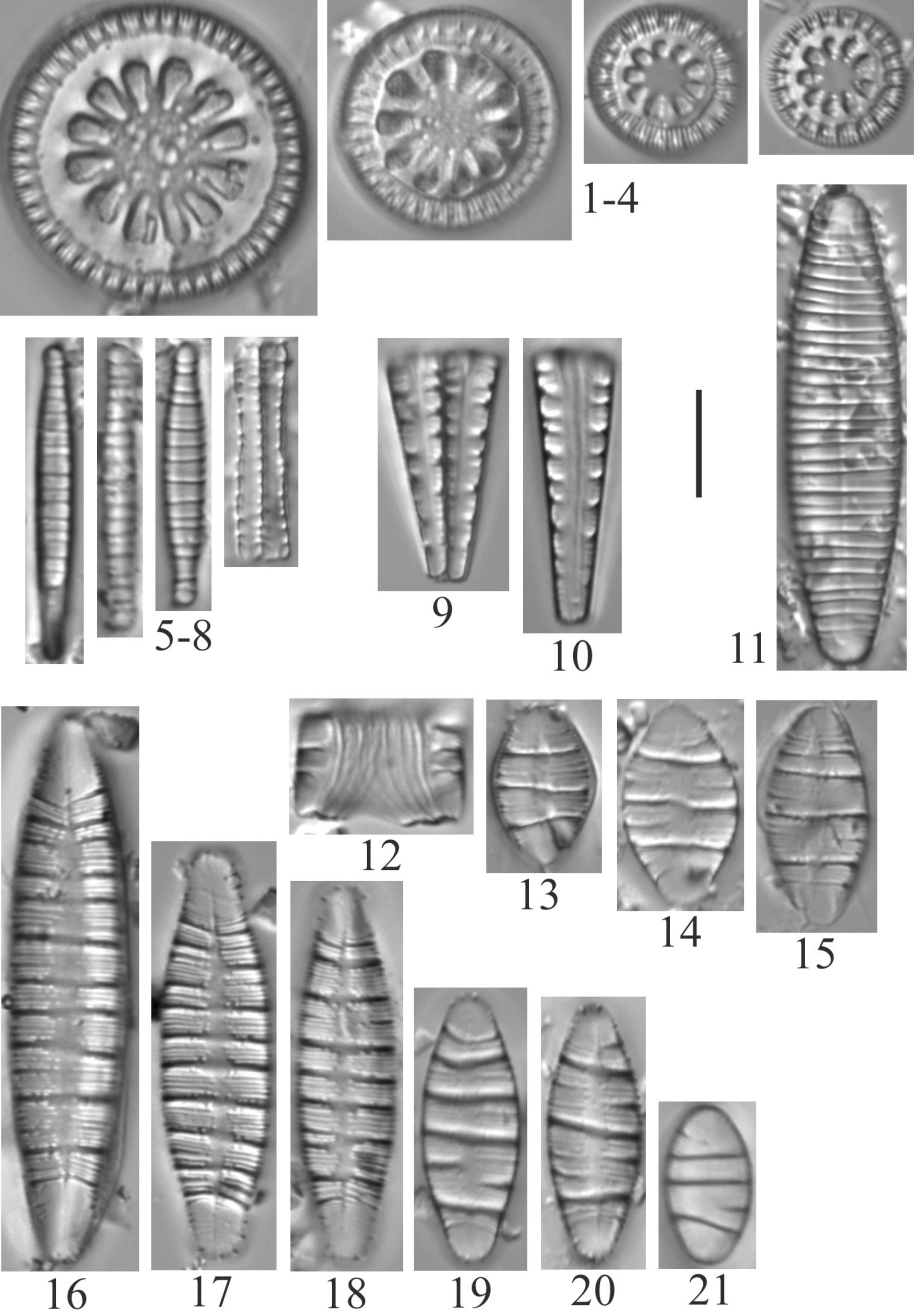
**1–4***Lindaviaantiqua* (6957) **5–8***Diatomatenuis*? [*D.moniliformis*?] (6958) **9, 10***Meridioncirculare* (6956) **11***Diatomavulgaris* (6955) **12–15***Odontidiummesodon* (6957) **16–21***Odontidiumhyemale* (6957). Scale bar: 10 µm.

**Plate 2.**
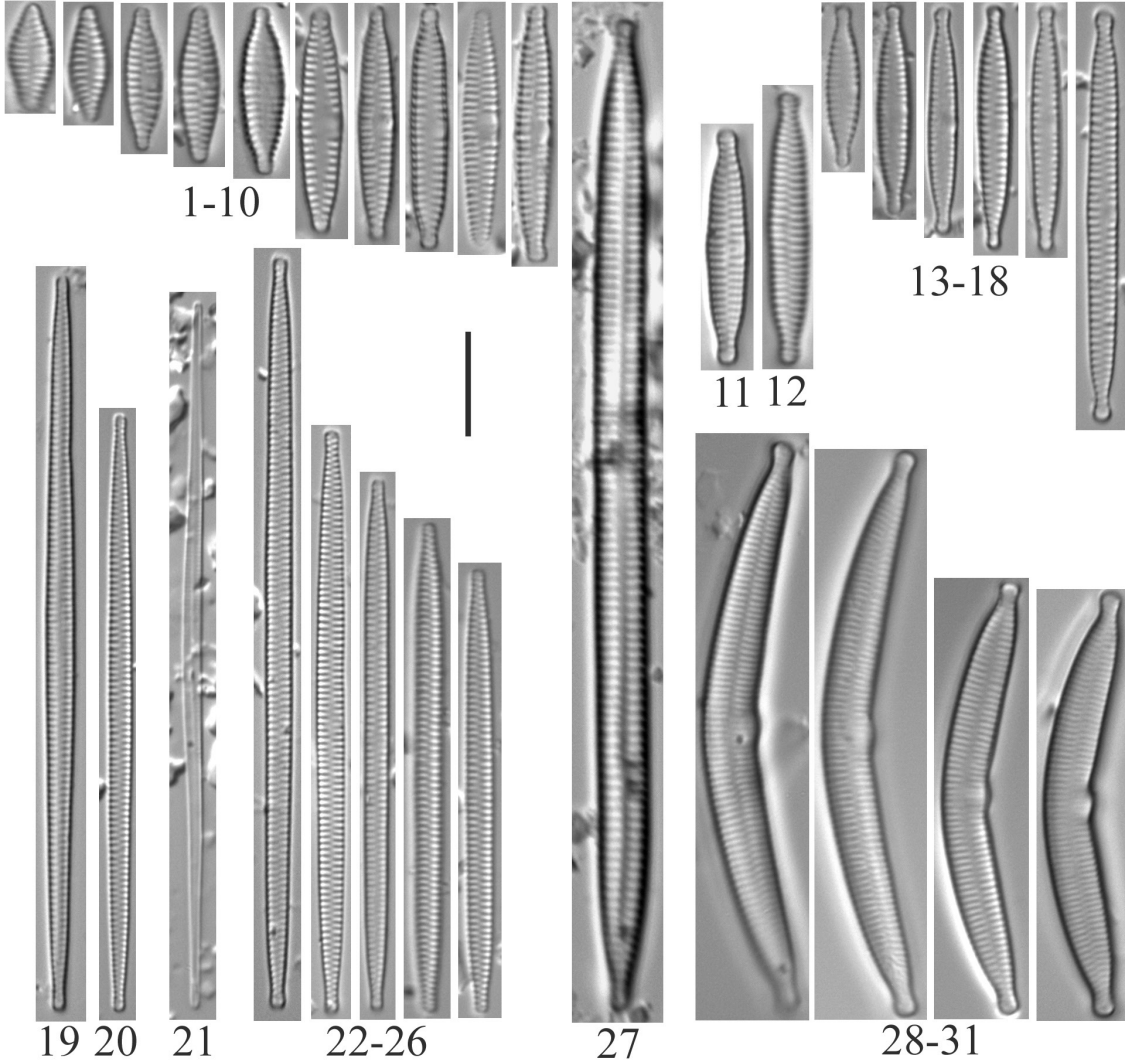
**1–10***Fragilariavaucheriae*? [*F.perminuta*?] (6957) **11, 12***Fragilaria* sp. (6956) **13–18***Fragilaria* sp. (6957) **19, 20***Fragilariatenera* (6956) **21***Fragilariasepes* (6955) **22–26***Fragilariaamphicephala* (6957) **27***Ulnariaulna*? (6958) **28–31***Hannaeaarcus* (6956). Scale bar: 10 µm.

**Plate 3.**
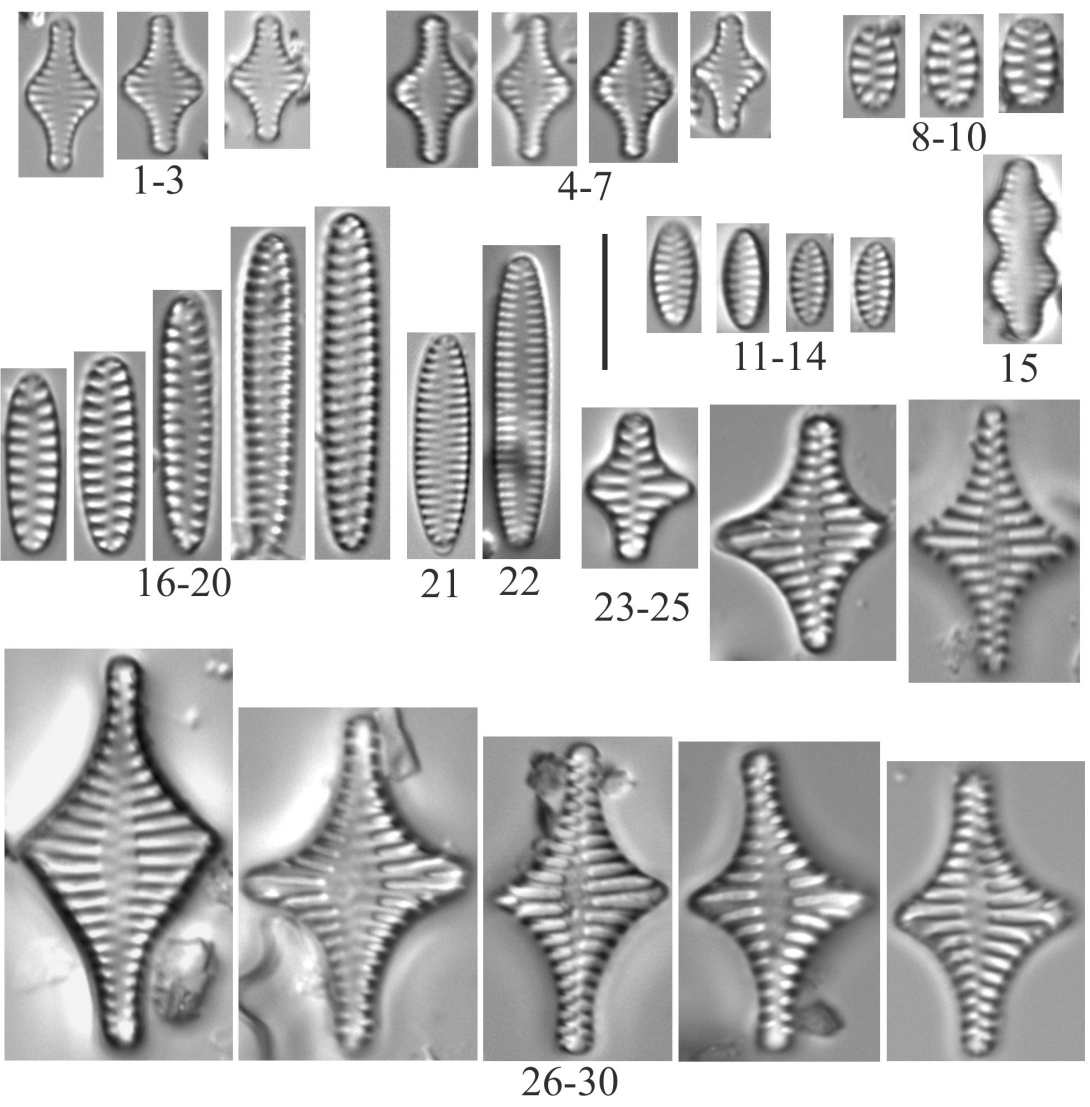
**1–7***Staurosiraconstruens* (6957) **8–10***Staurosirellapinnata* (6957) **11–14**Staurosirasp.cf.construensvar.venter (6957) **15***Pseudostaurosirarobusta* (6957) **16–20***Staurosirellalapponica* (6957) **21, 22***Staurosira* sp. (6957) **23–30***Staurosirellaleptostauron* (6957). Scale bar: 10 µm.

**Plate 4.**
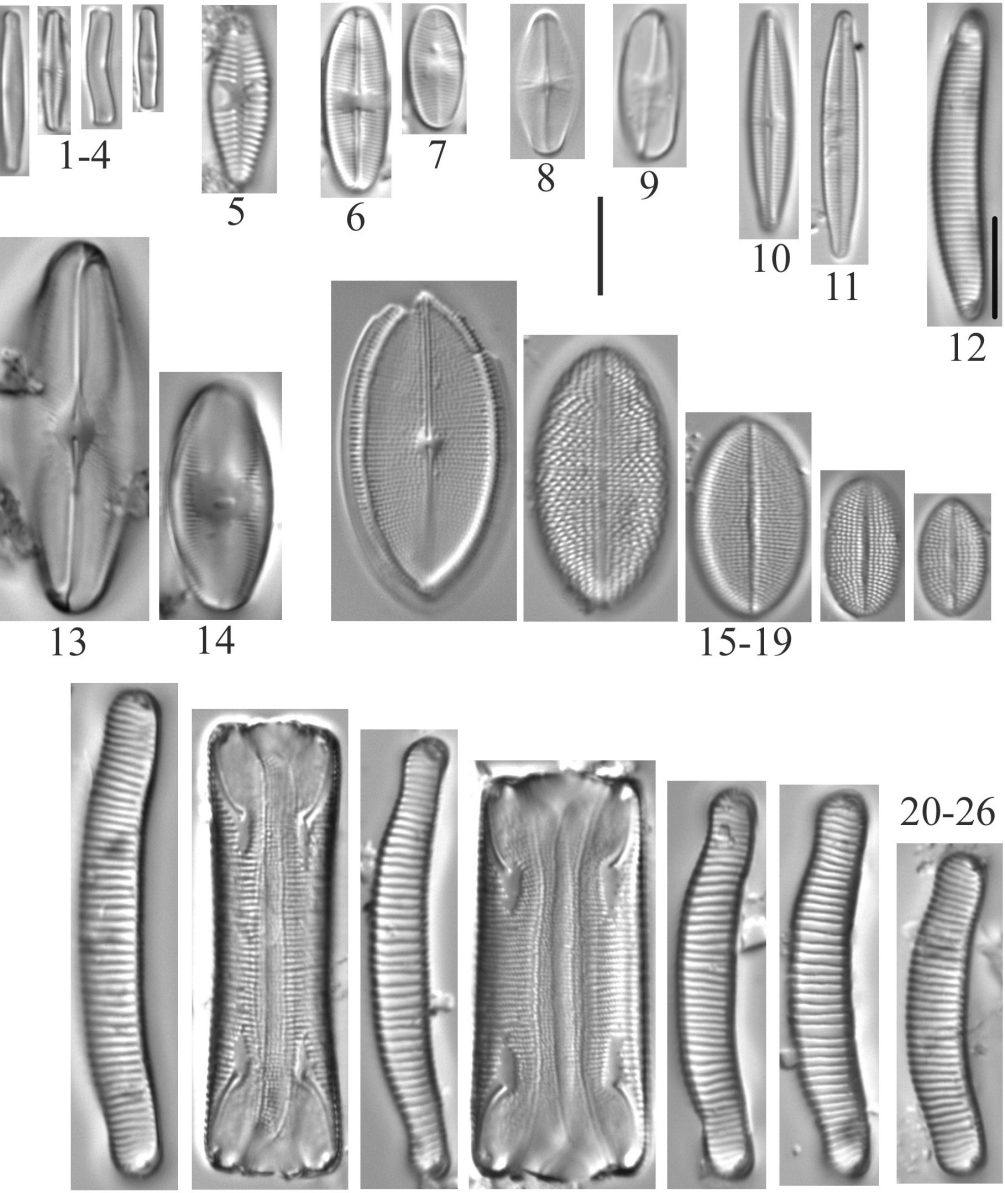
**1–4***Achnanthidiumminutissimum* (6955, 6956, 6957) **5***Planothidiumlanceolatum* (6958) **6, 7***Psammothidiumhelveticum* (6959) **8***Eucocconeislaevis* (6956) **9***Eucocconeisalpestris* (6956) **10, 11***Achnanthidiumgracillimum* (6956) **12***Eunotiavalida* (6956) **13, 14***Eucocconeisflexella* (6957) **15–19***Cocconeisplacentula* var.? (6956, 6957, 6958) **20–26***Eunotiaarcus* or *Eunotiaarcubus* Nörpel-Schempp & Lange-Bertalot (6957). Scale bar: 10 µm.

**Plate 5.**
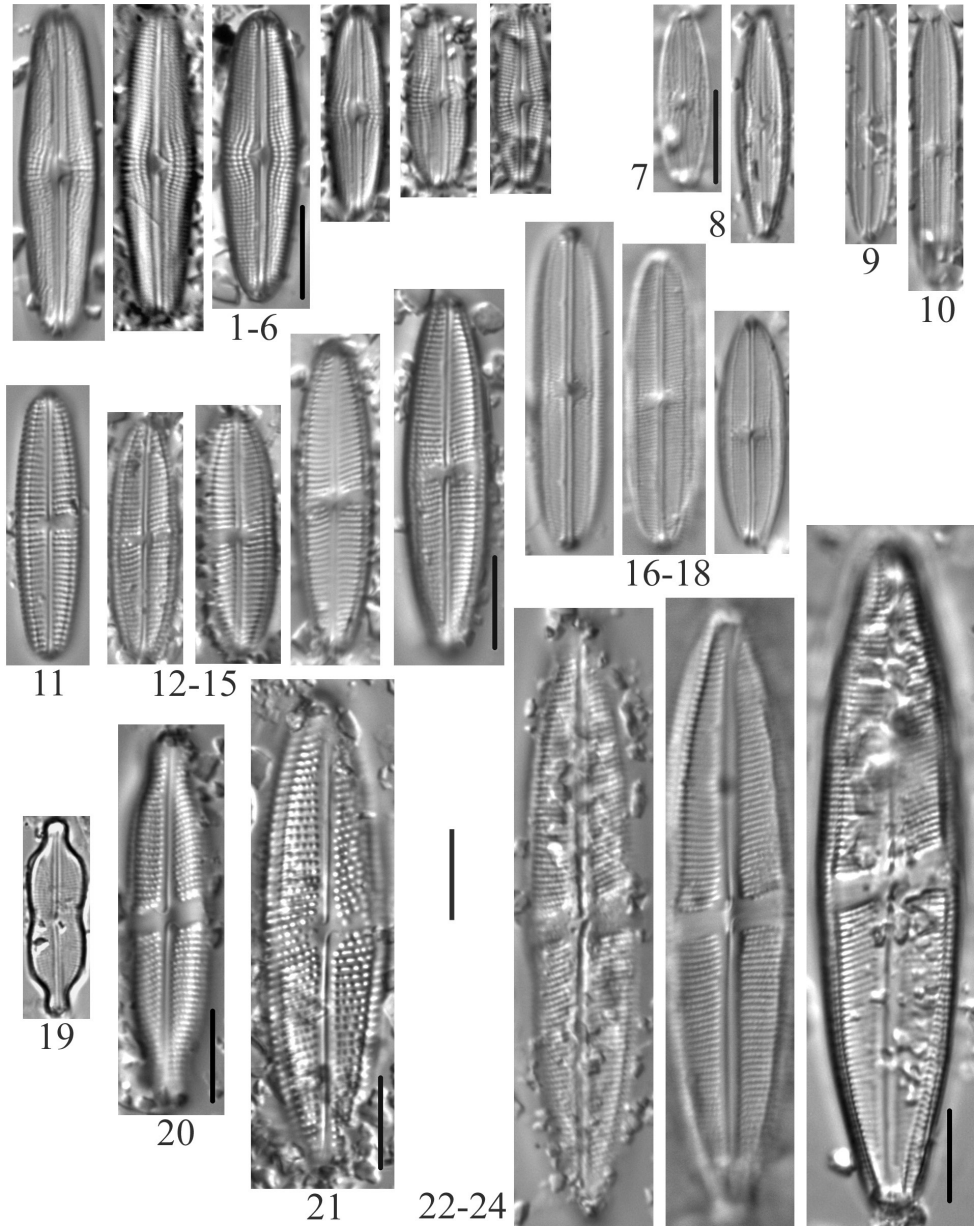
**1–6***Muelleriagibbula* (6955) **7, 8***Neidiumbergii* (6955) **9, 10***Neidium* sp. cf. *N.bisulcatum* (6955) **11***Neidium* sp. cf. *Neidiumboreale* Foged (6955) **12–15**Neidiumkozlowiivar.ellipticum (6955) **16–18***Neidium* sp. (6955, 6957) **19***Neidiopsisbinodiformis* (6957) **20***Neidiumfogedii* (6955) **21***Neidiumdistinctepunctatum* (6955) **22–24***Neidiumbobmarshallensis* (6955). Scale bar: 10 µm.

**Plate 6.**
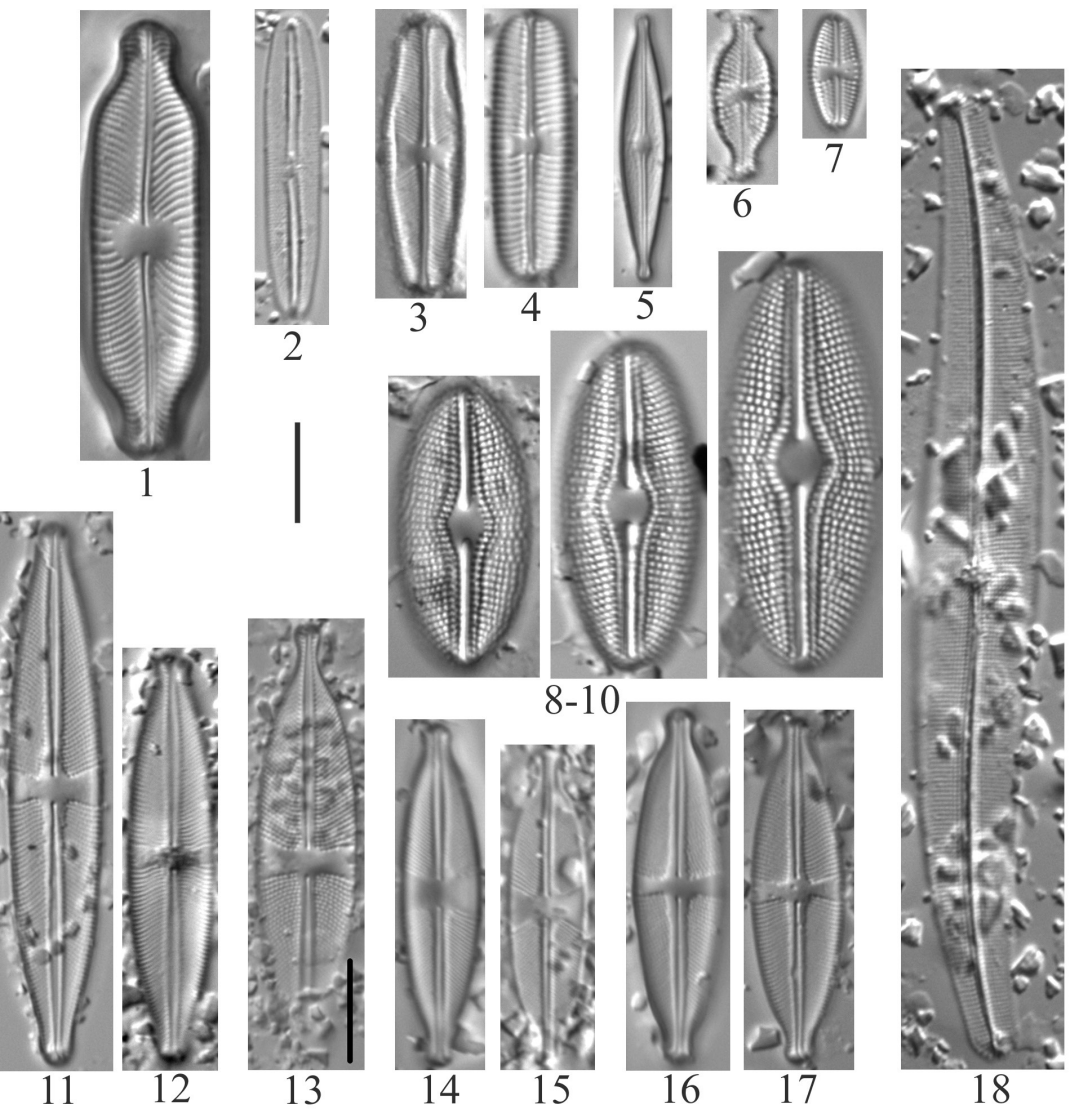
**1***Placoneisabiskoensis* Hustedt (6957) **2***Frustuliaamosseana* (6955) **3***Sellaphorapupula* (6957) **4***Sellaphoralaevissima* (6957) **5***Brachysiramicrocephala* (6956) **6***Luticolaventricosa* (6957) **7***Luticolamutica* (6957) **8–10***Diploneiskrammeri* (6957) **11, 12***Stauroneisgracilis* (6955) **13***Stauroneisamphicephala* or *Stauroneisancepsfallax* Bahls) (6955) **14, 15***Stauroneisvandevijveri* (*S. “arctic-anceps*” Van de Vijver et al.) (6955) **16, 17***Stauroneisreichardtii* (?) (6955) **18***Gyrosigma* sp. (6955). Scale bar: 10 µm.

**Plate 7.**
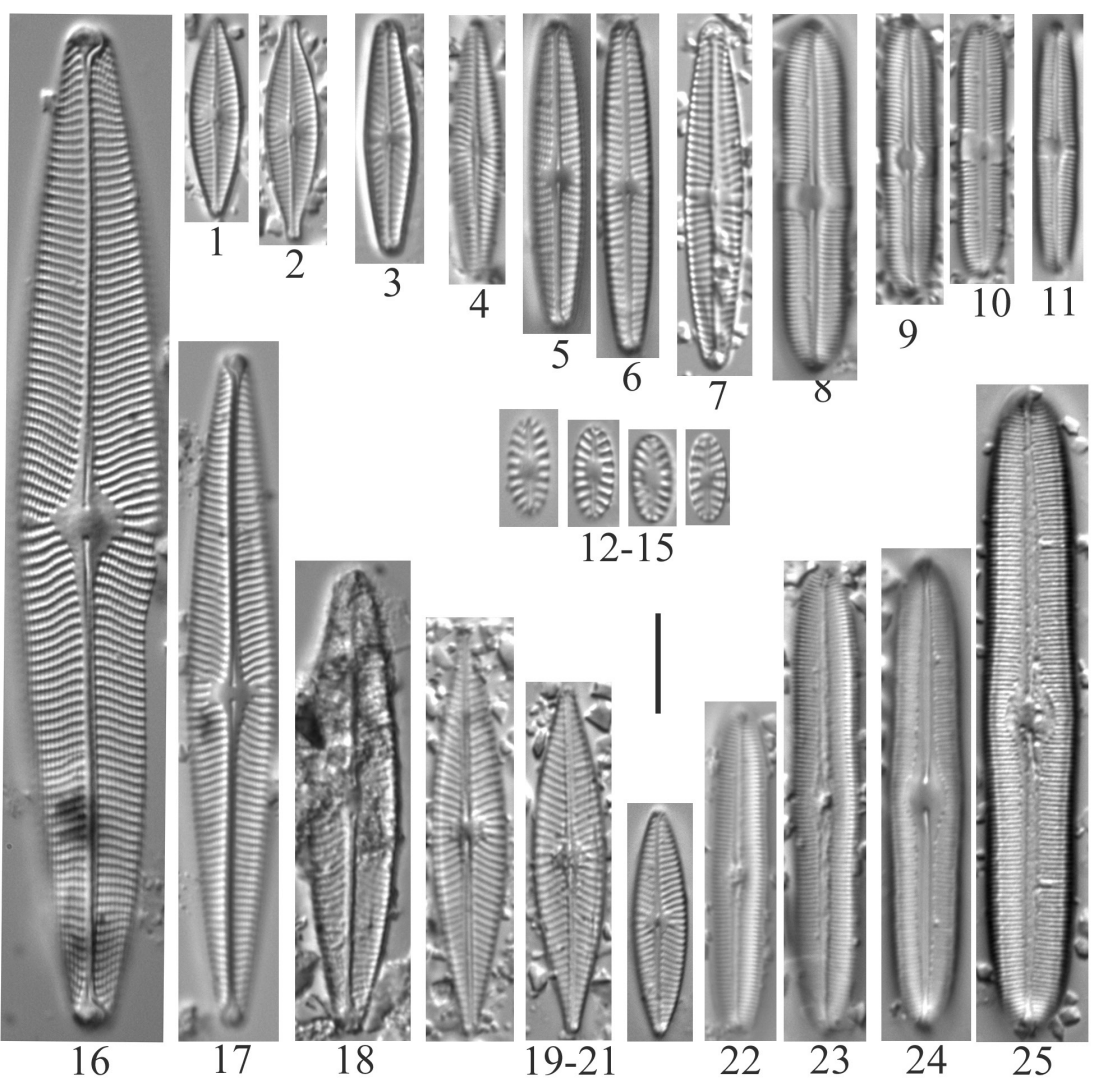
**1, 2***Naviculacryptocephala* (6955) **3***Naviculaseibigiana* (6956) **4***Naviculacryptotenella* (6955) **5, 6***Naviculaangusta* (6957, 6958) **7***Naviculalibonensis* (6955) **8***Caloneissilicula* (6957) **9, 10***Caloneisfalcifera* (6955) **11***Caloneistenuis* (6957) **12–15***Hygropetrabalfouriana* (6957) **16***Naviculavulpina* (6957) **17***Navicularadiosa* (6957) **18***Naviculalanceolata* (6958) **19–21***Naviculasubconcentrica* (6955, 6956) **22, 23***Caloneisthermalis* (6955) **24, 25***Caloneisalpestris* (6955, 6957). Scale bar: 10 µm.

**Plate 8.**
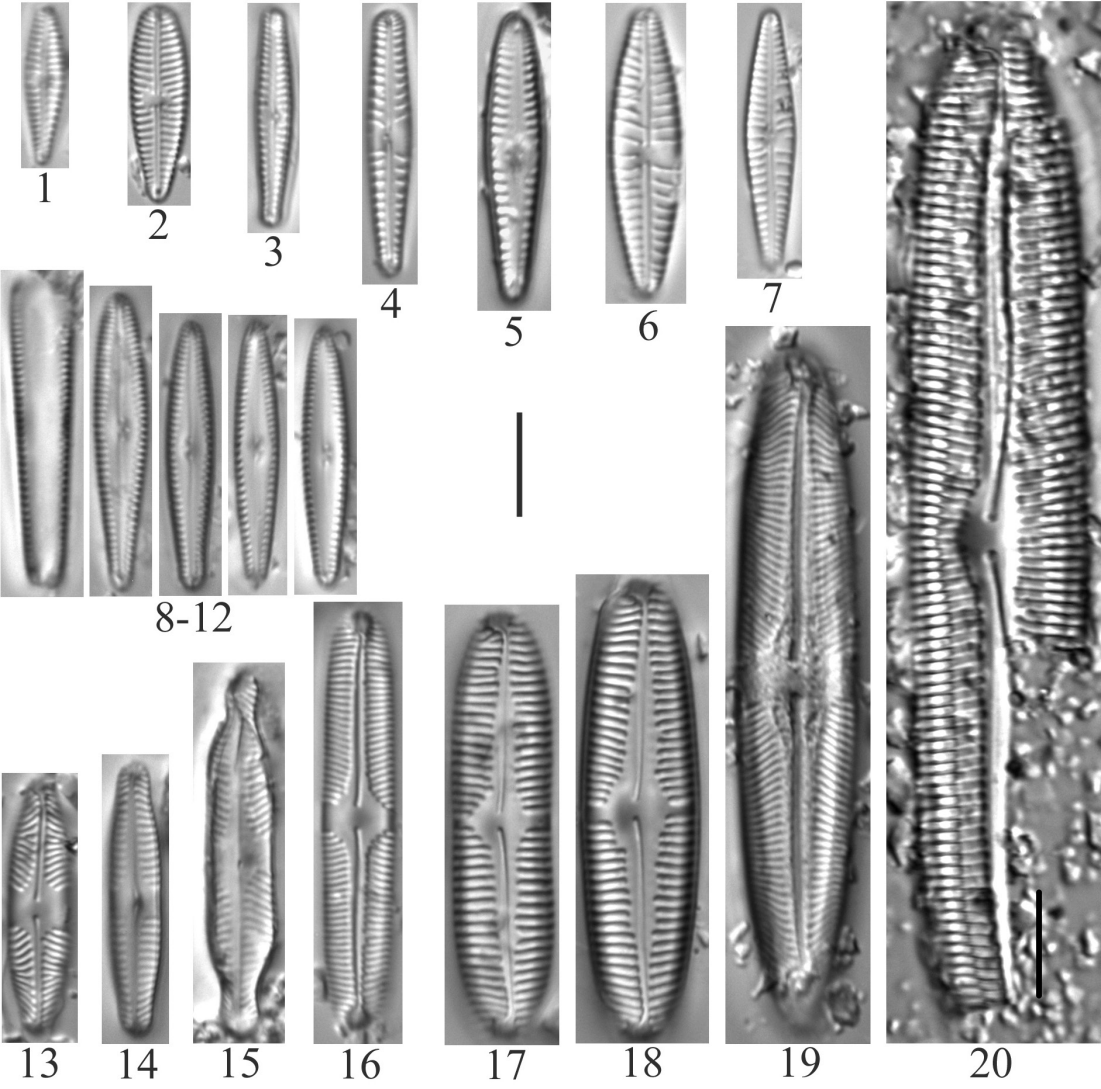
**1***Gomphonema* sp. (6957, 6959) **2***Gomphonemaolivaceoides* (6958) **3***Gomphonemaminusculum* Krasske (6958) **4***Gomphonemalateripunctatum* (6956) **5***Gomphonemapumilum*? (6956) **6***Gomphonemabozenae* (6957) **7***Gomphonemapseudobohemicum* (6958) **8–12***Gomphonemacaperatum* (6958) **13***Pinnulariakrammeri* (6955) **14***Pinnulariasinistra* (6956) **15***Pinnulariasubanglica* (6958) **16***Pinnularia* sp. (6957) **17, 18***Pinnulariasubcommutata* (6957) **19***Pinnulariapermicrostauron* (6955) **20***Pinnulariaviridis* (6955). Scale bar: 10 µm.

**Plate 9.**
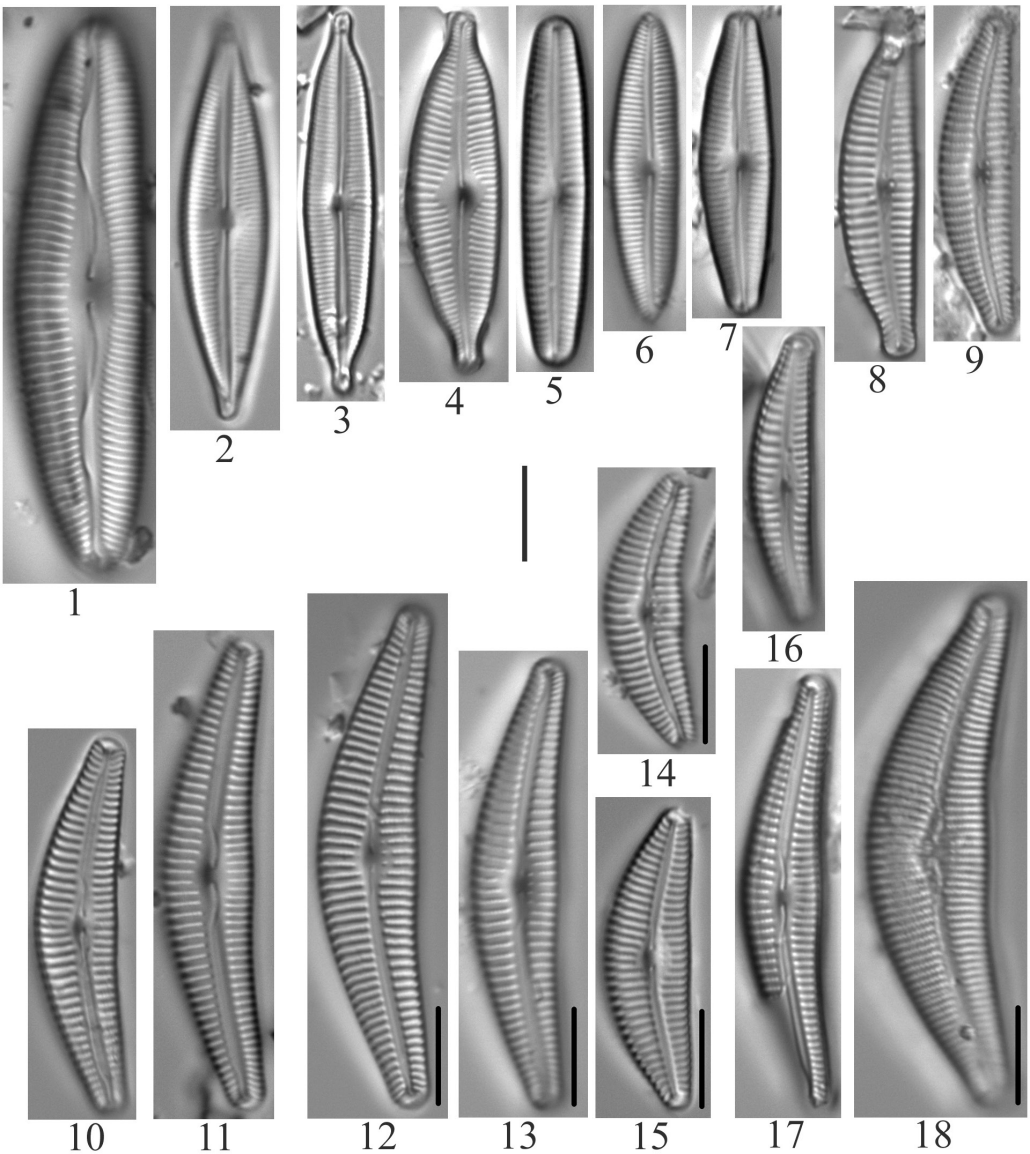
**1***Cymbopleuraaustriaca* (6957) **2***Cymbopleuralapponica* (6956) **3***Cymbopleuraangustata* (6958) **4***Cymbopleuranaviculiformis* (6959) **5***Cymbopleuraoblongata* (6956) **6***Cymbopleuraincerta* (6956) **7***Cymbopleurasubaequalis* (6958) **8, 9***Cymbellaexcisiformis* (6958) **10, 11***Cymbellaalpestris* (6957) **12–15***Cymbellaneocistula* (6956, 6957) **16***Cymbellacosleyi* (6956) **17***Cymbellacleve-eulerae* (6956) **18**Cymbellaneocistulavar.islandica (6956). Scale bar: 10 µm.

**Plate 10.**
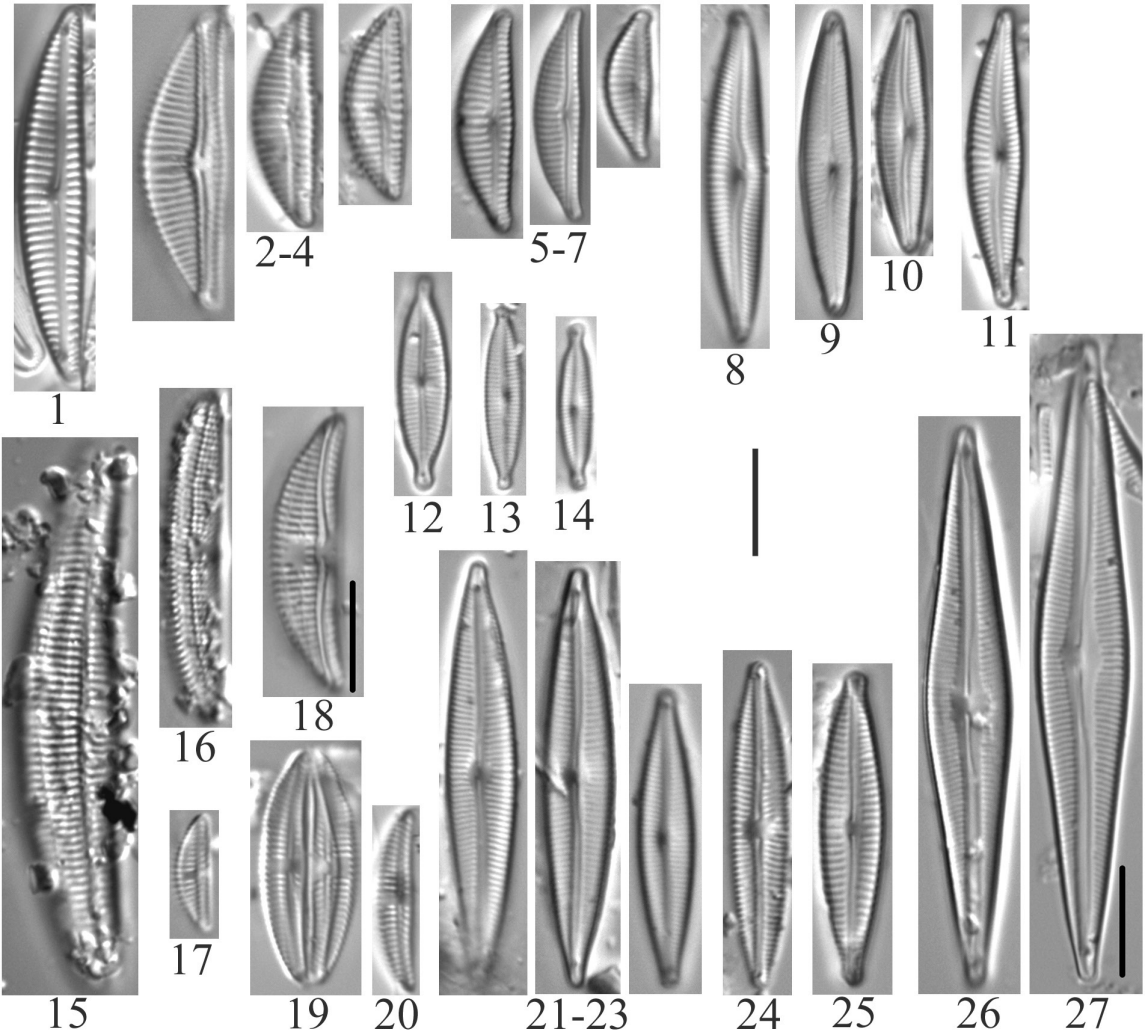
**1***Encyonemaneogracile* (6958) **2–4***Encyonemasilesiacum* (6957) **5–7***Encyonemaperminutum* (6956) **8***Delicatadelicatula* (6956) **9, 10***Delicataalpestris* (6956) **11***Delicata* sp. (6956) **12***Encyonopsisczarneckii* (6956) **13***Encyonopsissubminuta* (6956) **14***Encyonopsisalpina* (6956) **15, 16***Amphora* sp. (6955) **17***Amphorapediculus* (6957) **18***Amphoracopulata* (6956) **19, 20***Amphorainariensis* (6957) **21–23***Encyonopsisstafsholtii* (6956) **24***Encyonopsiscesatii* (6957) **25***Kurtkrammeriaaequalis* (6958) **26, 27***Encyonopsismontana* (6956). Scale bar: 10 µm.

**Plate 11.**
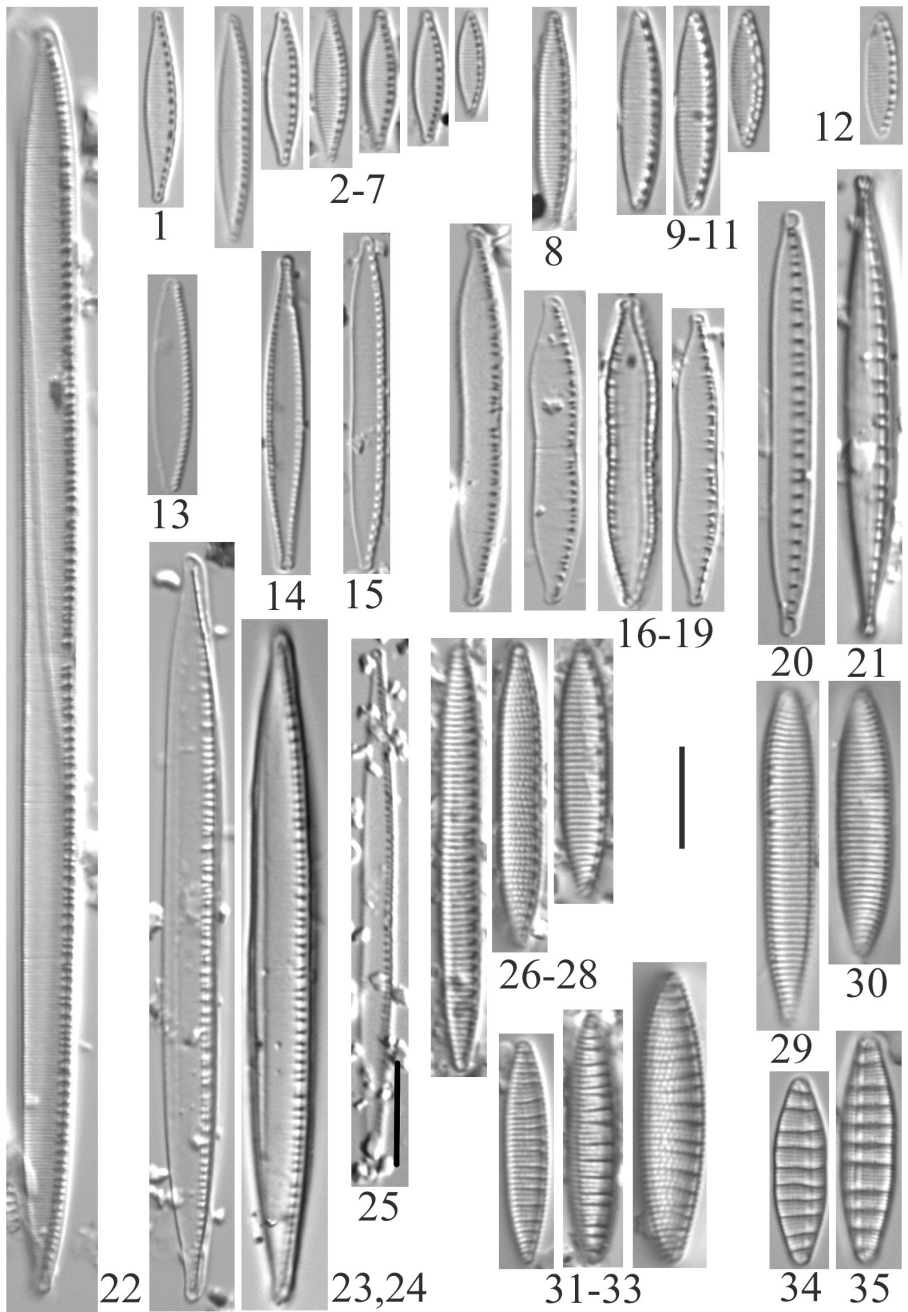
**1***Nitzschialacuum* (6956) **2–7***Nitzschiafonticola* (6957) **8***Nitzschiaperminuta* (6957) **9–11***Nitzschiaalpina* (6957) **12***Nitzschiainconspicua* (6957) **13***Nitzschiapusilla* (6956) **14***Nitzschiapura* (6956) **15***Nitzschiapalea* (6955) **16–19***Nitzschiahomburgiensis* (6957) **20***Nitzschiadissipata* (6957) **21**Nitzschiadissipatavar.oligotraphenta (6958) **22***Nitzschialinearis* (6957) **23, 24***Nitzschiasublinearis* (6956, 6957) **25***Nitzschiaexilis* (6955) **26–28***Nitzschiaamphibia* (6955) **29, 30***Nitzschiaangustata* (6957) **31–33***Denticulakuetzingii* (6955, 6957) **34, 35***Denticulatenuis* (6957). Scale bar: 10 µm.

**Plate 12.**
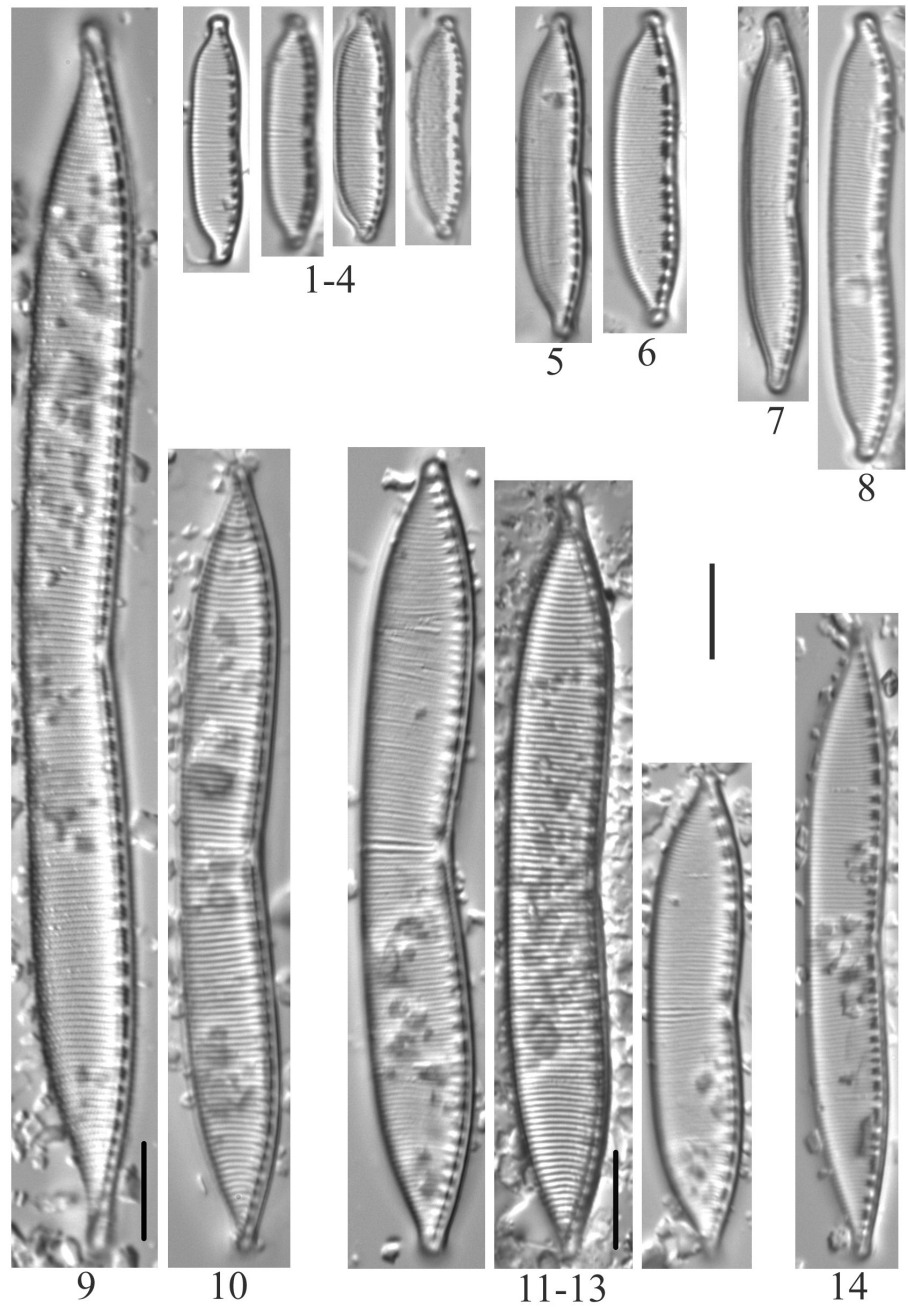
**1–4***Hantzschiaamphioxys* (6957, 6959) **5, 6***Hantzschia* sp. (6957) **7, 8***Hantzschiaabundans* [*Hantzschiaamphioxys*?] (6958) **9, 10***Hantzschiaelongata* (6955) **11–13***Hantzschiahyperborea* (6955) **14***Hantzschia* sp. (6955). Scale bar: 10 µm.

**Plate 13.**
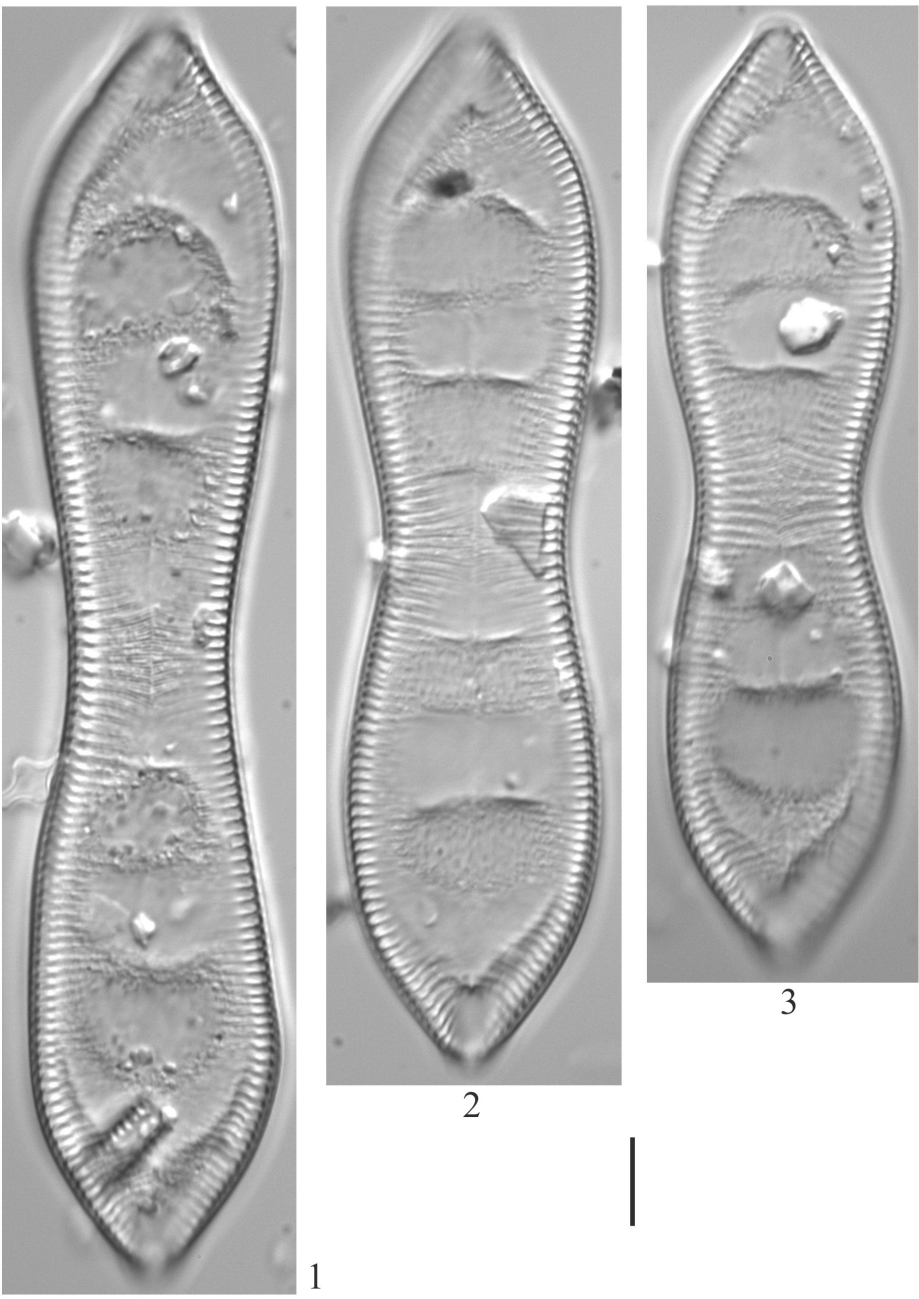
**1–3***Cymatopleurasolea* (6957). Scale bar: 10 µm.

**Plate 14.**
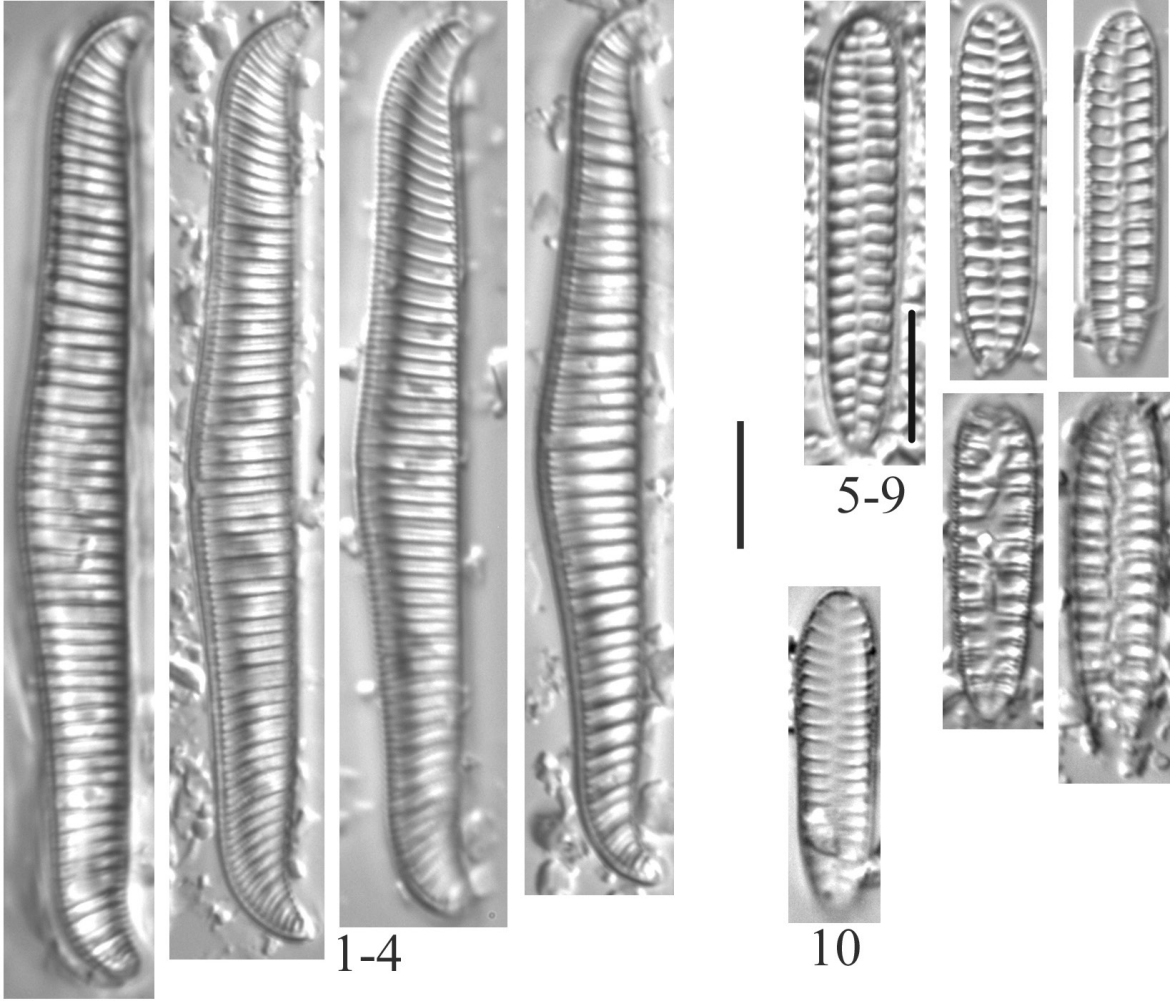
**1–4***Rhopalodiagibba* (6955) **5–9***Surirellaarctica* (6955) **10***Surirellaminuta* (6959). Scale bar: 10 µm.

## Discussion

McCarthy Spring had the highest species richness of the four habitats that were sampled, likely owing to the relative stability of this habitat compared to the lake, stream and glacier habitats. That most of the type localities for these taxa are in Europe is not surprising for two reasons: 1) Most of the early work in diatom taxonomy was conducted in Europe and 2) The existence of a Holarctic or circumboreal kingdom of diatoms has been well established (Bahls 2018). Only two of the species have their type localities in Alaska: *Neidiumfogedii* and *Surirellaarctica*.

Most of the 139 taxa documented from WRST are typical elements of diatom associations in the Northern Rocky Mountains of Alberta, Canada and Montana, USA (Bahls, unpublished data). Two notable exceptions from the Rocky Mountain flora are *Surirellaarctica* and *Gomphonemacaperatum*.

*Surirellaarctica* is a rare Arctic species that had been recorded previously only from localities in the high Arctic (Patrick and Freese 1961, Antoniades et al. 2008, Veselá and Potapova 2014, Veselá 2017). This is the first record of this species south of 68°N latitude. Its presence in WRST at 61°N latitude is likely made possible because of the extreme Arctic-like conditions that prevail in the park.

*Gomphonemacaperatum*, collected from a moulin on the Root Glacier, has a disjunct distribution in montane regions of the eastern and far western United States (Bishop 2017, Ponader et al. 2017). In the eastern U. S., it ranges from the southern Appalachians to Quebec (Ponader et al. 2017); in the West, it ranges from the Sierra Nevada Mountains in California up through the Willamette and Puget Sound basins of Oregon and Washington, respectively (Bishop 2017). Notably, it was the most common diatom on the Root Glacier and one of the few taxa from the glacier represented by more than one or two frustules, perhaps indicating a viable population in this habitat. This may be the first confirmed record of this taxon from Alaska.

The four samples reported here provide just a hint of the diatom biodiversity in this wild and immense national park. Samples from other lakes, springs, streams and glaciers and samples from other habitats (e.g. seeps and wetlands) will likely produce hundreds more taxa and provide more clues to the origins and geographic and ecological affinities of the WRST diatom flora. Elsewhere in Alaska, there are vast areas and countless diverse habitats that remain to be explored for diatoms.
